# Sensitive, Selective, and Fast Detection of ppb-Level H_2_S Gas Boosted by ZnO-CuO Mesocrystal

**DOI:** 10.1186/s11671-016-1688-y

**Published:** 2016-10-26

**Authors:** Yanan Guo, Miaomiao Gong, Yushu Li, Yunling Liu, Xincun Dou

**Affiliations:** 1 Laboratory of Environmental Science and Technology, Xinjiang Technical Institute of Physics & Chemistry, Key Laboratory of Functional Materials and Devices for Special Environments, Chinese Academy of Sciences, Urumqi, 830011 China; 2 University of Chinese Academy of Sciences, Beijing, 100049 China; 3 State Key Laboratory of Inorganic Synthesis and Preparative Chemistry, College of Chemistry, Jilin University, Changchun, 130012 People’s Republic of China

**Keywords:** Mesocrystal, p-n junction, Gas sensor, ppb, H_2_S detection

## Abstract

ZnO-CuO mesocrystal was prepared via topotactic transformation using one-step direct annealing of aqueous precursor solution and assembled into a H_2_S sensor. The ZnO-CuO mesocrystal-based sensor possesses good linearity and high sensitivity in the low-concentration range (10–200 ppb). Compared to pure CuO, the as-prepared ZnO-CuO mesocrystal sensor exhibited superior H_2_S sensing performance with a response ranging from 8.6 to 152 % towards H_2_S concentrations from 10 ppb to 10 ppm when applied at the optimized working temperature of 125 °C. The sensor showed excellent repeatability and good selectivity towards H_2_S gas even at a concentration four orders of magnitude lower than the interfering gases, such as H_2_, CO_2_, CO, NO_2_, acetone, and NH_3_. The improved sensitivity could be attributed partially to the effective diffusion of analyte gas through the mesocrystal surface and the abundant accessible active sites. Moreover, the nanoscale p-n junctions within the mesocrystal, which could effectively manipulate the local charge carrier concentration, are also beneficial to boost the sensing performance.

**Electronic supplementary material:**

The online version of this article (doi:10.1186/s11671-016-1688-y) contains supplementary material, which is available to authorized users.

## Background

H_2_S is generally produced as a by-product from petroleum refining, farming, and biogas production [1, 2]. As one of the most toxic and flammable gases, H_2_S affects the nervous system of human beings and can cause people to lose consciousness at very low concentrations [3]. The acceptable ambient limit for H_2_S (recommended by the Scientific Advisory Board on Toxic Air Pollutants, USA) is within the range of 20–100 ppb [4]. Besides, small amount of H_2_S in exhaled breath is usually used as a signaling molecule for metabolic disorder called as halitosis [5, 6]. Furthermore, H_2_S with a concentration as low as 10 ppb is known to deteriorate the performance of hydrogen fuel cells [7]. Therefore, there is a pressing need to explore efficient sensing devices with high sensitivity capable of detecting H_2_S at ppb level.

High response values have been achieved by using various metal oxide semiconductor sensing materials, including ZnO [8], In_2_O_3_ [9], CuO [10–12], SnO_2_ [13], and WO_3_ [14]. Among these reported semiconductors, CuO is especially favored in selective detection of H_2_S due to its p-type semiconducting property [15] and strong affinity towards H_2_S molecules [16]. The sensitivities are down to ppm and even sub-ppm levels; nevertheless, the response/recovery process is always quite long in the case of using individual metal oxide semiconductor. For example, CuO nanowire-based sensors are capable to detect H_2_S with a concentration as low as 10 ppb with a response of 4.8 %[11] and 30.9 %[12]; however, both the response/recovery times exceed 10 and 15 min, respectively. More seriously, a vertically aligned CuO nanowire array-based sensor is not recoverable when the concentration of H_2_S is higher than 1 ppm [10]. There are some reports to reduce the response/recovery time by incorporating CuO with ZnO to form ZnO-CuO composites (ZnO nanofiber [17], nanowire [18], nanorod [19, 20], hollow sphere [21] decorated with CuO nanoparticles and CuO-ZnO micro/nanoporous film [22]), and ZnO is used as the transducing material in all these cases. Nevertheless, these composite structures could only achieve the detection of H_2_S with a concentration from 500 ppb to 7 ppm. In addition, the working temperature for most of the reported H_2_S gas sensor is in the range of 150–450 °C. Thus, a structure with appropriate ZnO and CuO arrangement is highly desirable to boost the response value and to reduce the response/recovery time towards ppb-level H_2_S gas and, possibly, working at a relatively lower temperature.

Mesocrystal is an ordered superstructure formed through the oriented alignment of nanoparticles [23, 24]. The excellent electrical conductivity and abundant adsorption sites induced by the mesocrystal structure are exactly expected for gas detection [25]. So far, only a few research group realized the fabrication of mesocrystal gas sensors [26–28] and were based on one type of metal oxide nanoparticles. Recently, a unique ZnO-CuO mesocrystal was synthesized via topotactic transformation [29], and efficient charge transfer between n-type ZnO and p-type CuO nanoparticles was confirmed. It is expected that if this mesocrystal could be employed as a gas-sensing material, the sensing performance would be greatly benefited since the fundamental mechanism of chemiresistive gas sensing is the manipulation of charge transfer. Another prevailing advantage for this structure is the internal porosity [23], which allows both their outer and inner parts to participate in gas-sensing reactions, providing good diffusion and more accessible active sites, and thus a high response value. Nevertheless, this promising mesocrystal structure was largely ignored for ppb-level H_2_S gas sensing.

In this work, ZnO-CuO mesocrystal was prepared by merely using one-step direct annealing of aqueous precursor solution. The resulting gas sensor showed an enhanced sensing performance towards H_2_S even at a working temperature of 125 °C in terms of higher sensitivity, shorter response/recovery time in comparison with the pure CuO-based sensor and other nanostructure-based H_2_S sensors reported previously. The superior performance was attributed to a synergistic effect of the p-n junction built between ZnO and CuO, as well as the internal porosity for effective diffusion and adsorption induced by the unique structure of mesocrystal.

## Methods

### Preparation of ZnO-CuO Mesocrystal and Pure CuO

ZnO-CuO mesocrystal was prepared using a facile one-step direct annealing of aqueous precursor solution. In a typical procedure, polyethylene oxide/poly(p-phenylene oxide)/polyethylene oxide (P123, MW 5400, 0.104 g) was dissolved into deionized water (9.609 g) with stirring for 2.5 h, followed by the addition of Zn(NO_3_)_2_·6H_2_O (0.107 g) and Cu(NO_3_)_2_·6H_2_O (0.163 g). The mixture was stirred for 1.5 h, and NH_4_NO_3_ (0.636 g) was added to form a gel. The gel was stirred until it was homogeneous and then placed on a ceramic crucible to be treated under stage-temperature-programmed calcinations. The precursor solution was firstly heated from 300 to 525 K at a temperature ramp of 1.0 K/min and kept at 525 K for 40 min, followed by further heating to 775 k at a rate of 0.5 K/min and kept for 150 min at this temperature. Pure CuO was prepared using the same approach without adding Zn(NO_3_)_2_·6H_2_O.

### Material Characterization

X-ray diffraction (XRD) measurement was conducted using powder XRD (Bruker D8 Advance, with Cu-K_α_ radiation operating at 40 kV and 40 mA, scanning from 2*θ* = 30° to 80°). Field-emission scanning electron microscopy (FESEM, ZEISS SUPRA 55VP) and transmission electron microscope (TEM, JEM-2100) were used to characterize the morphology of the samples. N_2_ adsorption was performed at 77 K on NOVA 2200e (Quantachrome, USA), and the surface area data was calculated on the basis of the Brunauer-Emmett-Teller (BET) model. Inductively coupled plasma optical emission spectrometer (ICP-OES) analysis was carried out on PerkinElmer Optima 3300 DV.

### Gas-Sensing Performance Evaluation

Initially, the prepared material was mixed with deionized water in a weight ratio of 4:1 and ground in a mortar for 15 min to form a paste. The paste was then coated on a ceramic substrate by a thin brush to form a sensing film on which silver interdigitated electrodes with both finger-width and interfinger spacing of about 200 μm were previously printed. The thickness of the film was controlled by the brushed cycles. The sample was dried naturally in air overnight. The sensor was aged under 4V voltage at room temperature for about 24 h. Finally, the sensor was fixed on a microheater to control the working temperature through modulating the current. The required amount of gas was injected into the conical flask (1.2 L) using a syringe and was mixed with air (relative humidity (RH) was about 20 %). For gas-sensing test, the sensor was inserted into the target gas chamber and measured by a CGS-1TP intelligent gas-sensing analysis system (Beijing Elite Tech Co., Ltd., China). After the sensor resistance reached a new constant value, the sensor was then inserted into a same size conical flask full of air to recover. The relative sensor response in resistance is defined as, Response = (*R*_*g*_ − *R*_*a*_)/*R*_*a*_ × 100 %, where *R*_*g*_ and *R*_*a*_ are the electrical resistances of the sensor in target gas and in air. The response time is defined as the period in which the sensor resistance reaches 90 % of the response value upon exposure to the target gas, while the recovery time is defined as the period in which the sensor resistance changes to 10 % of the response value after the target gas is removed.

## Results and Discussion

**Fig. 1:**
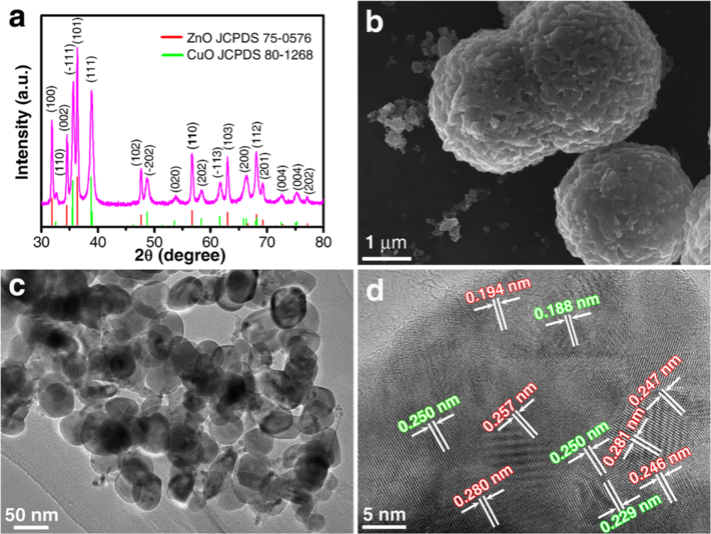
**a** XRD pattern. **b** SEM image. **c** TEM image. **d** HRTEM image of the ZnO-CuO mesocrystal

From the phase of the ZnO-CuO mesocrystal investigated by XRD (Fig. 1a), one can see clearly that the mesocrystal possesses a good crystallinity and there are two phases. One phase can be attributed to the CuO phase with a tenorite-type structure (JCPDS Card No. 80-1268), and the other one can be assigned to the ZnO phase with a zincite-type structure (JCPDS Card No. 75-0576). The broadening feature of the recorded peaks indicates that the sizes of the components are in the nanometer scale. According to the Scherrer relation: *D* = 0.9λ/*β*cos*θ*, where *D* is the average crystalline size, *θ* is the Bragg diffraction angle, and *β* is the full width at half maximum, the average nanocrystal size of ZnO is calculated to be 35 nm using the (100) and (101) peaks, and that of CuO is calculated to be 29 nm using the (−111) and (111) peaks. It is found that an annealing temperature of 250 °C is essential to achieve a topotactic transformation since intermediate phases of Zn(NO_3_)(OH)H_2_O and Cu_2_(OH)_3_NO_3_ exist when the annealing temperature is 200 °C (Additional file 1: Figure S1) and the reaction processes are summarized by Eqs. (1)–(4) in the supporting information. From the SEM image of the as-prepared ZnO-CuO mesocrystal (Fig. 1b), one can find that it is mainly composed of spheres with a size of about 1~2 μm assembled from nanoparticles. The detailed structure of the ZnO-CuO mesocrystal was further investigated by TEM. It is shown from the low-magnification image that the size of the composed nanocrystals is in the range of 25–40 nm (Fig. 1c), which is consistent with the XRD characterization. High-resolution transmission electron microscopy (HRTEM) image shows that the lattice spaces of 0.194, 0.257, 0.280, 0.281, 0.247, and 0.246 nm can be well corresponded to the (102), (002), (100), (100), (101), and (101) planes of zincite ZnO, respectively. And the lattice spaces of 0.188, 0.250, 0.250, and 0.229 nm correspond to the (−202), (−111), (−111), and (111) planes of tenorite CuO, respectively. Moreover, the similar crystalline lattices in the ZnO and CuO phases possess approximately the same crystallographic direction, indicating the successful preparation of the mesocrystal structure. It is worthy to note that the interface between ZnO and CuO can be clearly observed, without considerable interdiffusion, suggesting an abrupt p-n heterojunction interface formation. The formation of p-n junction creates a barrier for holes to transport in the valence band of CuO and provides a pathway for electrons in ZnO to transport through, which both locally lower the hole concentration in CuO and result in higher sensitivity to the gas molecule-induced charge transfer. Therefore, compared with that of bare CuO, the sensing performance of ZnO-CuO mesocrystal could be remarkably enhanced by the p-n junctions within the mesocrystal.

**Fig. 2:**
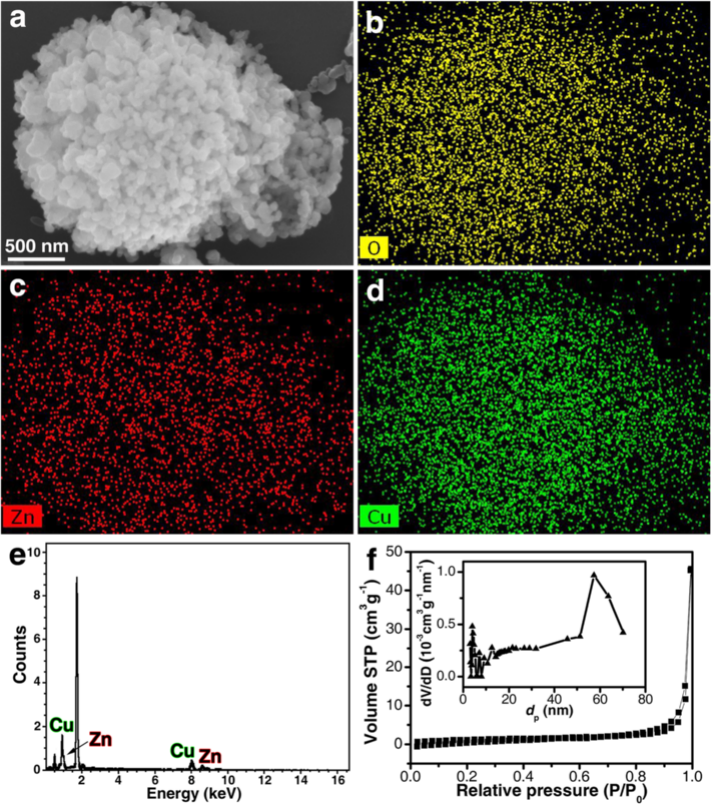
**a** SEM image, energy dispersive X-ray (EDX) elemental mapping of **b** O, **c** Zn, and **d** Cu of a ZnO-CuO mesocrystal, **e** a typical EDX spectrum of ZnO-CuO mesocrystal, and **f** N_2_ adsorption-desorption isotherm and pore size distribution (*inset*) of ZnO-CuO mesocrystal

Through the EDX elemental mapping of a ZnO-CuO mesocrystal (Fig. 2a), it is shown that elements O, Zn, and Cu are uniformly distributed in the mesocrystal (Fig. 2b–d). The atomic ratio of Cu:Zn determined by the EDX spectrum is 1.4:1 (Fig. 2e), which is consistent with the result of ICP-OES analysis (1.46:1). N_2_ adsorption analysis was performed to study the porosity of the ZnO-CuO mesocrystal. As shown in Fig. 2f, the curve is a type IV isotherm, and the BET surface area is about 10.21 m^2^/g and the pore diameter has a broad distribution with a predominant size of 57 nm. The pores within the ZnO-CuO mesocrystal provide direct diffusion channels for gas molecules to diffuse and contribute to fast adsorption/desorption at the interface, which is ideal for reducing the response/recovery time in gas sensing.

**Fig. 3:**
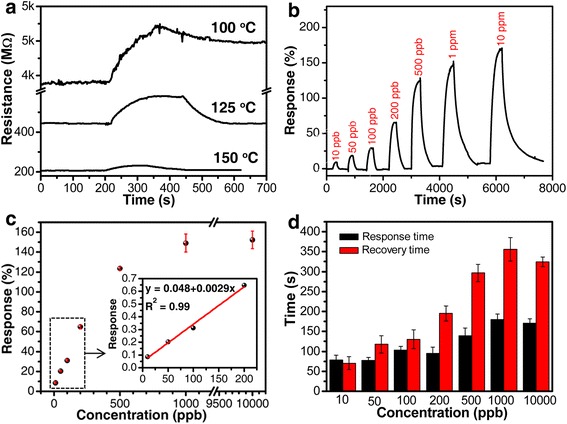
**a** Dynamic response curves of the ZnO-CuO mesocrystal-based sensor responding to 100 ppb H_2_S at different working temperatures, **b** plot of response versus time for a ZnO-CuO mesocrystal-based sensor upon exposure to H_2_S gas with concentrations ranging from 10 ppb to 10 ppm at 125 °C, **c** the corresponding calibration curve with error bar and a magnified image of the linear region (the *red line* is the linear fitting result), and **d** response time and recovery time with error bar

To determine the optimal operating temperature of the fabricated sensor with ZnO-CuO mesocrystal as the sensing layer, the sensing performance towards 100 ppb H_2_S at a series of operating temperatures from 100 to 150 °C was investigated. As shown in Fig. 3a, the sensor resistance increased fast upon exposure to H_2_S and then decreased to the initial resistance when the sensor was exposed to air. The typical p-type sensing behavior of the ZnO-CuO mesocrystal-based sensor with holes serving as the charge carriers confirmed that the signal transducing along the p-type CuO crystallites was dominant. The change in electrical resistance, as well as the recovery time, was strongly dependent on the working temperature. At operating temperatures of 100, 125, and 150 °C, the responses are about 20.6, 30.3, and 12.1 %, respectively. It is obvious that with the increase of the working temperature, the response of the sensor increases first and then decreases with a maximum value of 30.3 % at 125 °C. The reason for the lower response at 150 °C could be addressed to the weaker physical adsorption of H_2_S gas on the metal oxide surface and the lower concentration of the pre-adsorbed oxygen at high temperature. On the other hand, the recovery time is effectively shortened from several hours to less than 2 min by raising the operating temperature from 100 to 125 °C. Considering both the sensing response and recovery time, the optimized working temperature of the ZnO-CuO mesocrystal-based sensor was chosen as 125 °C for H_2_S detection.

To evaluate the sensitivity of the ZnO-CuO mesocrystal-based sensor, the typical dynamic response change measurements towards H_2_S with concentration ranging from 10 ppb to 10 ppm were conducted at 125 °C (Fig. 3b). The sensor showed apparent response of 8.6 % towards 10 ppb H_2_S. And with H_2_S concentration increasing to 10 ppm, the response of the sensor increased to 152 %. When the concentration is higher than 1 ppm, a saturation trend in response is observed, which might be resulted from the saturated adsorption of the active sites on the surface of the ZnO-CuO mesocrystal. Thus, the sensor is more suitable for the detection of ppb-level H_2_S. Figure 3c is the plot of the response of ZnO-CuO mesocrystal-based sensor as a function of H_2_S concentration with error bar. The trend roughly follows a Langmuir isotherm adsorption model as most commonly observed in chemiresistive sensors [30], a very fast linear increase in the low-concentration region, followed by a gentle slope in the high-concentration region. The capability of detecting low concentration of H_2_S (10–100 ppb) is critical in chemical diagnosis and quality control of industrial product. The linearity of the response values in the lower concentration range (10–200 ppb) possesses a slope of 0.0029 with a *R*-square value of 0.99 as shown in the inset of Fig. 3c. The limit of detection (defined as LOD = 3*S*_*D*_/*m*, where *m* is the slope of the linear part of the calibration curve (0.0029) and *S*_*D*_ is the standard deviation of noise in the response curve (1.6 × 10^−3^)) of the ZnO-CuO mesocrystal-based sensor is determined to be 1.7 ppb. The good linearity and low detection limit are in favor of the future device integration applied in detection of ppb-level H_2_S. Figure 3d presents the response/recovery time with error bar towards different concentrations of H_2_S, the response time keeps in the range of 78–180 s, while the recovery time increases from 70 to 356 s with the increase of the H_2_S concentration. The quick response and short recovery time should be attributed to the internal porosity of the ZnO-CuO mesocrystal, which could provide direct diffusion channels and facilitate the diffusion of H_2_S molecules, as well as fast adsorption/desorption at the interface.

**Fig. 4:**
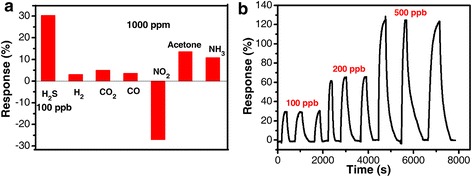
**a** Response of the ZnO-CuO mesocrystal-based sensor towards 100 ppb H_2_S and 1000 ppm H_2_, CO_2_, CO, NO_2_, acetone, and NH_3_ at 125 °C. **b** Response change of a sensor during three successive cycles of exposure to 100, 200, and 500 ppb H_2_S

For practical application, it is required that a gas sensor not only has superior sensor response and quick response/recovery process but also possesses good selectivity to the target gas. The selectivity of the ZnO-CuO mesocrystal-based sensor was then evaluated by comparing the responses of the sensor towards 100 ppb H_2_S and interfering gases, including H_2_, CO_2_, CO, NO_2_, acetone, and NH_3_ with a concentration of 1000 ppm at 125 °C (Fig. 4a). The response to 100 ppb H_2_S achieves 30.3 %, while the responses to 1000 ppm H_2_, CO_2_, CO, NO_2_, acetone, and NH_3_ are only 3.1, 5.1, 3.6, −27.1, 13.5, and 10.8 %, respectively, and the dynamic response curve is shown in Additional file 1: Figure S2. Thus, the sensor possesses a much higher response value to H_2_S even with a concentration four orders of magnitude lower than the other gases, implying that the ZnO-CuO mesocrystal-based sensor has very good selectivity towards H_2_S gas. It is known that the response of the metal oxide-based sensor would be remarkably affected by RH. Here, the responses of the sensor towards air with RH of 33, 54, 75, 85, and 95 % relative to air with RH of 20 % are evaluated to be 0, 7.4, 9.1, 11.1, and 22 %, respectively (Additional file 1: Figure S3). Thus, the response values of the present sensor towards H_2_S gas are not affected by humidity since the RH was 20 ± 2 % during the testing process. Furthermore, in order to ensure the repeatability of the present H_2_S gas sensor, it was exposed to 100, 200, and 500 ppb H_2_S for three successive cycles (Fig. 4b). The response value of each cycle keeps nearly the same, which are 30.3 ± 0.8, 64.8 ± 2.1, and 123.6 ± 1.8 %, respectively, indicating the excellent reproducibility of the present ZnO-CuO mesocrystal-based H_2_S sensor.

**Fig. 5:**
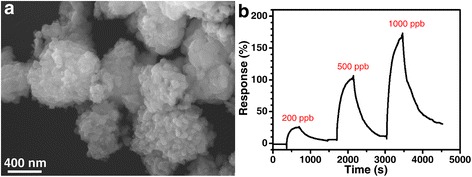
**a** SEM image of CuO microspheres. **b** Plot of response versus time for a CuO microsphere-based sensor upon exposure to 200 to 1000 ppb H_2_S gas at 125 °C

For comparison, the bare CuO with spherical morphology assembled with nanoparticles with a size of 50–60 nm was prepared via the same reaction procedure (Fig. 5a). The sensing performance of the CuO microspheres towards H_2_S was investigated, and the successive response-recovery sensing curve to various concentrations (200, 500, and 1000 ppb) of H_2_S at 125 °C was shown in Fig. 5b. Towards H_2_S with concentrations of 200, 500, and 1000 ppb, the response values of the CuO microsphere-based sensor are 24.7, 100.7, and 160.8 %, which are comparable to those of the ZnO-CuO mesocrystal-based sensor with 64.8, 123.6, and 158.1 %, respectively. However, the sensitivity of pure CuO-based sensor is much lower than that of the ZnO-CuO mesocrystal-based sensor since it has no apparent response to 100 ppb H_2_S. Moreover, the response/recovery times of CuO are within the range of 130–230 and 503–1034 s, respectively, which are much longer than those of the ZnO-CuO mesocrystal. Thus, the ZnO-CuO mesocrystal with internal p-n junctions has an obvious advantage in ppb-level H_2_S sensing in terms of higher sensitivity and shorter response/recovery time.

The overall performance of the ZnO-CuO mesocrystal-based sensor is also excellent in comparison with the previous reports on CuO or CuO-ZnO-based H_2_S gas sensors, as shown in Table 1. The present sensor performance is superior to that of a CuO nanowire-based sensor with a response of 5 % towards 10 ppb H_2_S [11]. In addition, although the other sensors possess much higher responses, they are either applied in ppm-level H_2_S sensing or working at higher temperature. It is clearly shown that the ZnO-CuO mesocrystal-based sensor possesses a superior sensing performance towards H_2_S since the detection concentration of 10 ppb is the lowest, and the response value reaches 10 % at a moderate working temperature (125 °C).

**Table 1:** Various CuO and ZnO-CuO nanostructures employed for H_2_S sensing (C is the lowest tested concentration)

Sensing material	C (ppb)	Temperature (°C)	Response (%)	Response time (s)	Recovery time (s)	Ref.
CuO nanowire	10	180	5 %	600	1000	[11]
ZnO-CuO composite nanofiber	1000	150	3000 %	–	–	[17]
CuO-ZnO composite hollow sphere	1000	336	600 %	300	900	[21]
CuO nanoparticle decorated ZnO nanorod	10,000	100	3800 %	125	180	[33]
CuO/ZnO heterostructured nanorod	6750	500	7000 %	200	90	[19]
Ultrathin CuO layers modified ZnO nanowire	500	200	200 %	260	150	[18]
CuO-ZnO micro/nanoporous film	500	225	2000 %	35	80	[22]
CuO-ZnO powder	50,000	108	2000 %	–	–	[34]
Network CuO-ZnO composite	100	225	200 %	50	130	[35]
ZnO-CuO mesocrystal	10	125	10 %	78	70	This work

**Fig. 6:**
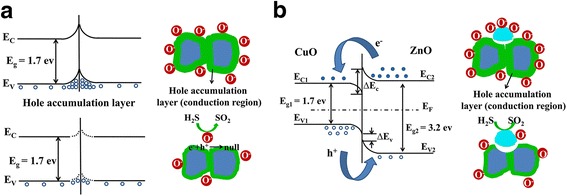
The energy band diagrams and schematic models of **a** CuO and **b** CuO-ZnO p-n junction before and after exposure to H_2_S gas. *blue* CuO, *cyan* ZnO

The operating principle of CuO-based gas sensor is based on the change of the sensor conductivity by controlling the mobility of the charge carriers, and the working principles in air and in the target gas are schematically shown in Fig. 6a. In air atmosphere, oxygen molecules adsorb on the surface of CuO in the form of O_2_^−^ (below 100 °C) and O^−^ (100–300 °C), serving as electron acceptors by capturing the electrons in the conduction band of CuO [31]. The transfer of electrons from CuO to oxygen species results in a local hole accumulation layer at the surface of CuO. In the presence of a reducing gas, for example, H_2_S, electrons generated from the redox reactions between H_2_S and the chemisorbed surface oxygen anions will be injected back to the conduction band of CuO. The recombination between electrons and holes will result in a lower hole concentration, thus induce an increase of resistance.

However, the sensing mechanism of the ZnO-CuO mesocrystal is different. The electrons in ZnO and holes in CuO diffuse in opposite direction due to the great gradient of the same carrier concentration until the diffusion and drift of the carriers are finally balanced. Consequently, p-n junction is formed, and the energy band bends in the depletion layer to achieve a uniform Fermi level (E_F_) in the thermal equilibrium state (Fig. 6b). It can be calculated that the barrier height of the conduction band (Δ*E*_*c*_ = *E*_*c*2_ − *E*_*c*1_) and the valence band (Δ*E*_*v*_ = (*E*_*g*2_ − *E*_*g*1_) − Δ*E*_*c*_) at the p-n junction are 0.77 and 0.73 eV, respectively [32]. Therefore, compared with that of bare CuO, the sensitivity of ZnO-CuO mesocrystal for the detection of H_2_S can be enhanced in two ways: (1) the hole concentration in CuO can be lowered not only by the electron transfer from the H_2_S gas molecule (elector donor) but also by the electron injection from the conduction band of ZnO, leading to an increase in resistance; (2) the electron concentration in ZnO gets higher due to the electron transfer from H_2_S to ZnO, which renders a stronger p-n junction, thus helps to block the local hole transportation around the junction region in CuO and yields a further increase in resistance.

## Conclusions

In summary, the ZnO-CuO mesocrystal was prepared via topotactic transformation using one-step direct annealing of aqueous precursor solution. The as-prepared sensor exhibited superior H_2_S sensing performance, such as low detection limit, high response, and fast response/recovery process. Moreover, the sensor showed excellent repeatability and good selectivity towards H_2_S gas even at a concentration four orders of magnitude lower than the interfering gases, including H_2_, CO_2_, CO, NO_2_, acetone, and NH_3_. The enhanced response of the ZnO-CuO mesocrystal to H_2_S is attributed partially to the effective diffusion of H_2_S molecules through the entire porous surface and the formation of uniform nanoscale p-n junction within the mesocrystal. Thus, the ZnO-CuO mesocrystal represents a promising sensing material for sensitive and selective detection of ppb-level H_2_S gas.

## Additional file

Additional file 1: Figure S1. XRD patterns of the products obtained from zinc nitrate and copper nitrate as the mixed precursor by annealing at 200 and 250 °C. **Figure S2.** Dynamic response curves of the ZnO-CuO mesocrystal-based sensor responding to 1000 ppm NO_2_, H_2_, CO_2_, CO, acetone and NH_3_ at 125 °C. **Figure S3.** The responses of the ZnO-CuO mesocrystal based sensor upon exposure to air with different relative humidity relative to air with RH of 20 % at 125 °C. (DOCX 198 kb)
