# Supplementary material for: Sensitive, Selective, and Fast Detection of ppb-Level H2S Gas Boosted by ZnO-CuO Mesocrystal
Source: Nanoscale Res Lett. 2016 Oct 26;11:475. doi: 10.1186/s11671-016-1688-y (PMC5081309; doi:10.1186/s11671-016-1688-y)
Supplement: Additional file 1: Figure S1. — XRD patterns of the products obtained from zinc nitrate and copper nitrate as the mixed precursor by annealing at 200 and 250 °C. Figure S2. Dynamic response curves of the ZnO-CuO mesocrystal-based sensor responding to 1000 ppm NO2, H2, CO2, CO, acetone and NH3 at 125 °C. Figure S3. The responses of the ZnO-CuO mesocrystal based sensor upon exposure to air with different relative humidity relative to air with RH of 20 % at 125 °C. (DOCX 198 kb) [file 11671_2016_1688_MOESM1_ESM.docx]

Supporting information

**Sensitive, Selective and Fast Detection of ppb-level H_2_S Gas Boosted by ZnO-CuO Mesocrystal**

*Yanan Guo^a,*^,* *Miaomiao Gong ^a,b^, Yushu Li ^a^,* *Yunling Liu^c^ and Xincun Dou^a,*^*

^a^ Laboratory of Environmental Science and Technology, Xinjiang Technical Institute of Physics & Chemistry; Key Laboratory of Functional Materials and Devices for Special Environments, Chinese Academy of Sciences, Urumqi 830011, China

^b^ University of Chinese Academy of Sciences, Beijing 100049, China

^c^ State Key Laboratory of Inorganic Synthesis and Preparative Chemistry, College of Chemistry, Jilin University, Changchun 130012, P.R. China

* Correspondence: guoyn@ms.xjb.ac.cn, xcdou@ms.xjb.ac.cn

**Reactions** **in the one-step direct annealing process:**

$Zn(NO_{3})_{2}+2H_{2}O\to\mathrm{Zn}\left( NO_{3} \right)\left( \mathrm{OH} \right)H_{2}O+HNO_{3}$ (1)

$\mathrm{Zn}\left( NO_{3} \right)\left( \mathrm{OH} \right)H_{2}O\to ZnO+HNO_{3}+H_{2}O$ (2)

$2Cu(NO_{3})_{2}+3H_{2}O\to Cu_{2}(OH)_{3}(NO_{3})+3HNO_{3}$ (3)

$Cu_{2}(OH)_{3}(NO_{3})\to2CuO+HNO_{3}+H_{2}O$ (4)





Figure S1. XRD patterns of the products obtained from zinc nitrate and copper nitrate as the mixed precursor by annealing at 200 and 250 ^o^C.


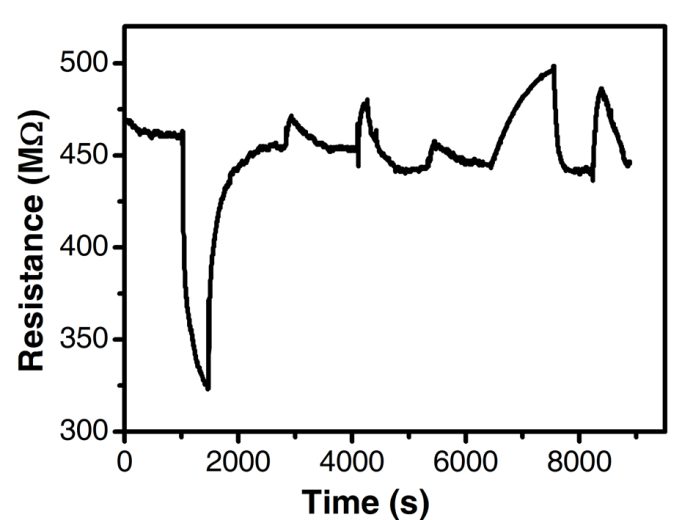


Figure S2. Dynamic response curves of the ZnO-CuO mesocrystal-based sensor responding to 1000 ppm NO_2_, H_2_, CO_2_, CO, acetone and NH_3_ at 125 °C.


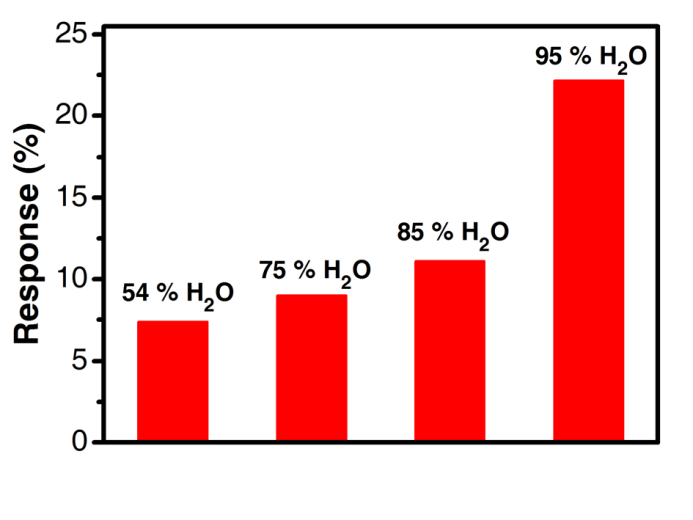


Figure S3. The responses of the ZnO-CuO mesocrystal based sensor upon exposure to air with different relative humidity relative to air with RH of 20% at 125 ^o^C.
